# HCV RNA quantification using the Cobas Plasma Separation Card as an alternative to EDTA plasma: a prospective multicenter study

**DOI:** 10.1038/s41598-026-45854-x

**Published:** 2026-04-20

**Authors:** Ana Fuentes López, Ariadna Rando Segura, Raquel Carracedo, Aline Kessler, Adolfo de Salazar González, Sirak Zenebe Gebreab, Esther Molina, Noelia Parajó, Sara Pereira, Judit Romero Vico, Elena Ruiz Escolano, Marta Vila, Laura Viñuela González, Ana Carrasco Durán, Antonio Aguilera, Federico García, Maria Buti

**Affiliations:** 1 Hospital Universitario San Cecilio, Granada, Spain; 2 Ciber de Enfermedades Infecciosas CIBERINFEC, Instituto de Investigación Biosanitaria Ibs.Granada, Granada, Spain; 3 Hospital Universitari Vall d’Hebron, Barcelona, Spain; 4 Centro de Investigación Biomédica en Red de Enfermedades Hepáticas y Digestivas (CIBERehd), Instituto de Salud Carlos III, 28029 Madrid, Spain; 5 Fundación Instituto de Investigación Sanitaria de Santiago de Compostela, Santiago de Compostela, Spain; 6 Hospital Clinico Universitario de Santiago, Santiago de Compostela, Spain; 7 Roche Diagnostics International Ltd, Rotkreuz, Switzerland

**Keywords:** Clinical performance, Dried blood spot, Dried plasma sample, Hepatitis C virus, RNA quantification, Viral load, Diseases, Gastroenterology, Medical research, Microbiology

## Abstract

Simplified diagnostic approaches are needed to eliminate hepatitis C by 2030. This prospective multicenter study evaluated dried plasma spots collected with the Cobas® Plasma Separation Card (PSC) versus conventional ethylenediaminetetraacetic acid (EDTA) plasma for hepatitis C virus (HCV) RNA detection and quantification using Cobas HCV (nucleic acid amplification test). Cobas PSCs were spotted with finger prick-collected capillary (PSC capillary) or venous (PSC venous) blood from individuals with known HCV infection status. HCV infection status was based on anti-HCV antibodies and HCV RNA in EDTA plasma. The threshold for clinical sensitivity/specificity was 1000 IU/mL RNA. Quantitative HCV RNA results were compared between EDTA plasma and Cobas PSC samples. Among 224 subjects, 119 had undetectable and 105 had detectable HCV RNA. Clinical sensitivity and specificity versus EDTA plasma were 100% for PSC capillary, and 97.8% and 100%, respectively, for PSC venous. HCV viral loads from PSC capillary and PSC venous were comparable with EDTA plasma. Cobas PSC combined with Cobas HCV showed good diagnostic accuracy versus EDTA plasma; quantitative HCV RNA results were comparable between the two sample types. Cobas PSC sample collection may obviate the need for venipuncture and help to broaden the reach of HCV testing in remote or under-resourced regions.

**Supplementary Information:**

The online version contains supplementary material available at 10.1038/s41598-026-45854-x.

## Introduction

Hepatitis C virus (HCV) can cause acute and chronic hepatitis, with the latter ranging from mild disease to liver cirrhosis and hepatocellular carcinoma^1,2^. The World Health Organization (WHO) estimated that in 2022, 50 million individuals globally were affected by chronic HCV infection, with an estimated 242,000 deaths; approximately 1.0 million new infections occur annually^1,3^. HCV infection represents a significant global health problem due to its associated morbidity and mortality^4^. A disproportionate HCV disease burden exists in low‐ and middle‐income countries^5^.

The WHO has established HCV elimination targets, aiming to reduce HCV-associated mortality by 2030, via improvements in HCV prevention, diagnosis, and treatment^3^. Underdiagnosis is a key barrier to achieving this goal, with many individuals remaining unaware of their infection because they are asymptomatic or experience non-specific HCV symptoms^1^. Improved early diagnosis of HCV infection is key for reducing onward transmission and improving outcomes by facilitating timely treatment, which helps to prevent disease progression^1,6,7^. The WHO has also highlighted the need for better linkage between HCV diagnosis and provision of care^3^.

To diagnose HCV infection, a two-step approach is recommended by international clinical practice guidelines by organizations such as the WHO, European Association for the Study of the Liver (EASL), American Association for the Study of Liver Diseases (AASLD), Infectious Diseases Society of America (IDSA), the Centers for Disease Control and Prevention (CDC)^6–9^. Using this approach, individuals are firstly tested for anti-HCV antibodies in serum or plasma via enzyme immunoassay; if anti-HCV antibodies are detected, HCV RNA or HCV core antigen should be determined to diagnose active infection^6–9^. HCV RNA assays are the preferred approach for confirming diagnosis, as HCV core antigen assays are less sensitive for the diagnosis of viremia^8,9^. EASL recommends detection of HCV RNA in serum or plasma using a sensitive, qualitative, or both qualitative and quantitative, molecular method with a lower limit of detection (LLOD) of ≤ 15 IU/mL^8^. However, to broaden access to HCV diagnosis and care in settings where sensitive HCV RNA assays are unavailable or unaffordable, less sensitive methods may be considered; WHO and EASL consider an LLOD of ≤ 1000 IU/mL (3.0 log_10_ IU/mL) HCV RNA acceptable for the identification of viremic HCV infection, for the purpose of HCV diagnosis, or assessment of treatment response^8,9^. A low incidence of false negative results is expected with this higher RNA threshold^8,10^.

Despite advances in HCV treatment (e.g., potentially curative direct-acting antivirals), there are numerous challenges facing HCV care, including healthcare access relating to HCV diagnosis and logistical issues with sample storage and transport^10,11^. Furthermore, a systemic approach is lacking for testing and screening, for reaching isolated communities, and laboratory infrastructure^11^. The WHO recommends simplification of the care pathway to overcome barriers in access to HCV testing and treatment, especially in decentralized settings and among vulnerable or marginalized populations worldwide (e.g., people who inject drugs [PWID]) and people in prison^9,12^.

Dried blood spots (DBS), comprising whole blood samples collected on filter paper, are an alternative specimen type to EDTA plasma that can help increase access to HCV testing and linkage to care^13^. DBS do not require venipuncture, are suitable for serological and nucleic acid testing in both centralized and decentralized diagnostic settings, may be used to expand access to diagnosis in remote and under-resourced regions, and are suitable for large epidemiological surveillance studies^12,14,15^. Importantly, storage and transport of DBS do not require refrigeration and therefore enable sample stability under harsh environmental conditions^11,13^. International clinical practice guidelines, including those from EASL, AASLD, IDSA, and WHO recommend DBS as an alternative matrix to collect and transport samples for detection of anti-HCV antibodies and reflex HCV RNA testing^7–9,12,15^. However, DBS approaches are limited by having a lower sensitivity for HCV RNA quantification than serum or plasma^16–18^ and a variable quantitative performance depending on storage conditions^18,19^.

The Cobas® Plasma Separation Card (PSC) is a CE-approved collection device that was initially approved for HIV RNA testing^20,21^. Studies have also reported using Cobas PSC for anti-HCV antibody testing and HCV RNA quantification^14,22–26^. In December 2024, the Cobas PSC received CE-mark approval for use with Cobas HCV (on the Cobas 5800/6800/8800 systems). This approval validated dried plasma spots collected with the Cobas PSC as a sample type for the detection and quantification of HCV RNA in HCV-infected individuals. The PSC offers several advantages over DBS, including a porous membrane with RNA-stabilizing reagent that allows passive generation of a dried plasma spot from whole blood (140 µL of whole blood for each of three spots is the approved volume for HIV and HCV RNA quantification, as per the manufacturer’s instructions)^27^, and facilitates sample storage/transportation in extreme heat and humidity^14,20^. Head-to-head studies comparing HCV RNA quantification between Cobas PSC and DBS are lacking. However, a Ugandan study in a limited-access setting reported high correlation between HIV viral load results from venous Cobas PSC and plasma EDTA, and lower viral load results from DBS versus Cobas PSC or plasma EDTA^28^.

The clinical performance of dried plasma spots collected with Cobas PSC, in combination with Cobas HCV (a CE- and FDA-approved nucleic acid amplification test [NAAT] for the detection and quantification of HCV RNA), has been investigated in several small, single-center studies^14,22–24,26^. The aim of this multicenter prospective study was to evaluate the clinical utility of Cobas PSC in combination with the Cobas HCV test for HCV RNA detection and quantification, using capillary or venous blood samples in comparison with EDTA plasma to diagnose viremic HCV infection.

## Patients and methods

### Study design

Recruitment of consenting HCV-positive and HCV-negative individuals was conducted at three sites in Spain (Barcelona, Granada, Santiago de Compostela) between July 2023 and May 2024. This study design and methods complied with the International Council for Harmonisation Good Clinical Practice guidelines, the Declaration of Helsinki, all applicable laws in Spain including data privacy, as well any other relevant national/international standards/regulations. The study protocol was approved by the relevant ethics committee at each participating site (Comité De Ética De La Investigación Con Medicamentos for Fundació Hospital Universitari Vall d’Hebron; Comité De Ética De La Investigación Biomédica De La Provincia De Granada for Hospital Universitario San Cecilio; Comité De Ética De La Investigación Con Medicamentos De Galicia for Foundation Health Research Institute of Santiago de Compostela [IDIS]). The study was also approved by the Spanish Agency of Medicines and Medical Products (AEMPS). Written informed consent was obtained from each subject prior to their enrollment in the study.

### Study population and sample collection

Eligible subjects were 18 years or older, with positive or negative HCV infection status based on their medical history (i.e., self-reported or physician-reported suspicion or confirmation). Collection and processing of samples were performed at all three study sites. Subjects provided 10 mL venous blood collected via venipuncture in an EDTA tube for generation of EDTA plasma. Cobas PSCs were spotted with capillary blood collected via finger prick (PSC capillary) and a second Cobas PSC was spotted using venous blood obtained via venipuncture (PSC venous); whole blood (140 μL) was spotted on each of the three spots. Samples were stored according to the assay manual and transported to the testing site for processing within 24 h of blood draw.

### HCV status

HCV infection status—active or no active HCV infection—was determined based on the presence or absence of HCV antibodies and HCV RNA in EDTA plasma. Antibodies to HCV were measured using a CE-marked assay (Elecsys® Anti-HCV II [Roche Diagnostics], Alinity I Anti-HCV Assay [Abbott] or ARCHITECT Anti-HCV Assay [Abbott]). HCV quantification was conducted with the Cobas HCV assay using the Cobas 6800 system according to the manufacturer’s instructions. Detectable HCV RNA results were defined using an HCV RNA assay with cutoff of 15 IU/mL (based on EASL recommendations)^8^.

### Site training

 All study personnel were trained onsite (by the Sponsor team) on the protocol, the manual of procedure(s), and study documentation. During site initiation, site collection personnel (i.e., nurses) were trained on collection of capillary blood (in particular, ensuring a firm finger prick to obtain a sufficient volume of blood), and the appropriate use of Cobas PSC consumables for spotting the Cobas PSC (in particular, correct handling of the capillary and dispenser to prevent clots and to ensure full transfer to the PSC). The study team responsible for the pre-analytics were experienced laboratory personnel who received training on sample handling, pre-analytics and testing. In all three sites, the samples were tested by routine operators who used the instruments on a regular basis. To ensure site personnel were comfortable with study-specific sample collection, processing, and workflows, a study familiarization phase was performed after completion of training and prior to collection of study data.

### Technical performance of the Cobas PSC

Studies to assess sample stability and determination of limit of detection (LOD) and lower limit of quantification (LLOQ) were conducted by Roche Diagnostics International Ltd. as part of internal analytical performance evaluation studies. These analytical studies were conducted in parallel with the clinical study as detailed in the Supplementary methods online.

For the interpretation of PSC results from the clinical study, LOD and LLOQ values from these internal analytical technical studies (Supplementary methods online) were applied, including an LOD of 534.4 IU/mL (95% confidence intervals [CI]: 460.0, 648.3) and LLOQ of 879 IU/mL (Supplementary Table S1 online), representing a quantification linear range of 8.80E + 02 to 1.0E + 08 IU/mL for Cobas HCV when combined with Cobas PSC. The stability results from these internal studies demonstrated that Cobas PSC samples were stable for 28 days’ transport at 18–45 °C and up to 85% humidity, followed by 56 days’ storage stability with and without layer separation at: ≤  − 10 °C, 2–8 °C, and 18–30 °C (Supplementary Table S2 online).

### Statistical analysis

All samples with valid test results from at least one Cobas PSC sample type and EDTA plasma were included in the data analysis.

The comparability between quantitative HCV RNA results obtained from PSC capillary and venous sample types, and those obtained from EDTA plasma samples, was evaluated based on the accuracy of HCV RNA measurements and the consistency of bias across the measurement range. The analysis used only detectable Cobas HCV results (above the LLOQ) that were within the overlapping linear measurement range (15 IU/mL to 1.00E + 08 IU/mL for EDTA plasma and 880 IU/mL to 1.00E + 08 IU/mL for PSC capillary/venous). Bland–Altman analysis was performed to assess how closely the measurements of paired PSC and EDTA plasma sample results matched. For each paired sample, the differences between the PSC and EDTA plasma results were calculated and plotted against the average of the paired results. The mean paired difference, corresponding 95% CI, and the 95% limits of agreement were determined. Additionally, Deming regression analysis was conducted to evaluate the relationship between the different sample types (PSC capillary/venous and EDTA plasma) and to identify potential systematic differences between paired-sample results. A regression line was fitted to the paired data, and the model parameters (intercept and slope), along with their 95% CIs and the coefficient of determination (R^2^) were calculated. All analyses were conducted using log_10_-converted HCV RNA concentrations. The same analyses were conducted to compare results from PSC capillary and PSC venous sample types.

The diagnostic accuracy of PSC capillary/venous sample types was assessed at the HCV RNA threshold of 1000 IU/mL, using EDTA plasma as the reference sample matrix. Clinical sensitivity was calculated as the percentage of samples with an HCV viral load result of ≥ 1000 IU/mL in EDTA plasma that were correctly identified using PSC capillary/venous samples. The clinical specificity was calculated as the percentage of samples with an HCV viral load result of < 1000 IU/mL in EDTA plasma that were correctly identified using PSC capillary/venous samples. Positive predictive value (PPV) was calculated as the percentage of samples with an HCV viral load of ≥ 1000 IU/mL in PSC capillary/venous that truly have an HCV viral load of ≥ 1000 IU/mL in EDTA plasma, and negative predictive value (NPV) was calculated as the percentage of samples with an HCV viral load of < 1000 IU/mL in PSC capillary/venous that truly have an HCV viral load of < 1000 IU/mL in EDTA plasma. Additionally, total percent agreement was calculated as the proportion of samples where the PSC capillary/venous overall results matched the reference sample matrix. To provide a range of plausible estimate values, the 95% CIs of all parameters were calculated using the Wilson ‘score’ method^29^.

The SAS/STAT statistical analysis software package was used for all data analyses and summaries.

## Results

### Study population

A total of 299 subjects were enrolled in the study, from whom 293 EDTA plasma, 292 PSC capillary, and 293 PSC venous samples were collected; 224 EDTA plasma, 223 capillary, and 224 venous samples were used for data analysis, with reasons for sample exclusion presented in Supplementary Table S3 online. The demographic characteristics of the study population are provided in Table 1 and medical history in Supplementary Table S4 online.

**Table 1:** Baseline demographic and clinical characteristics of study population. One enrolled subject who withdrew from the study did not provide any demographic information. The analysis population included samples that contributed to at least one of the study objectives; one individual was not included in the analysis population for PSC capillary vs. EDTA plasma comparison; age was not reported for five subjects. HCV, hepatitis C virus; SD, standard deviation.

Characteristics	Enrolled population	Analysis population
Total, N	299	224
Age (years)		
Mean (SD)	49.4 (15.1)	49.3 (15.2)
Median, (range)	50.0 (20, 91)	50.0 (20, 91)
Gender, *n* (%)		
Male	171 (57.2)	121 (54.09)
Female	127 (42.5)	103 (46.0)
Race, *n* (%)		
Asian	3 (1.0)	3 (1.3)
Black or African American	3 (1.0)	2 (0.9)
Native Hawaiian or Other Pacific Islander	0 (0.0)	0 (0.0)
White	292 (97.7)	219 (97.8)
Ethnicity, *n* (%)		
Hispanic or Latino	187 (62.5)	154 (68.8)
Not Hispanic or Latino	111 (37.1)	70 (31.3)
Anti-HCV therapy received, *n* (%)		
No	197 (65.9)	163 (72.8)
Yes	50 (16.7)	13 (5.8)
Unknown	41 (13.7)	41 (18.3)
Not reported	10 (3.3)	7 (3.1)

Among the analysis population of 224 subjects, 119 had undetectable and 105 had detectable HCV RNA (LLOQ 15 IU/mL) in EDTA plasma. HCV viral load was between 15 and 880 IU/mL for 12 subjects, and between 1000 and 1E + 08 IU/mL for the remaining 93 HCV-positive EDTA plasma samples (Supplementary Table S5 online).

### Comparison of quantitative Cobas HCV results between sample types

#### PSC capillary and EDTA plasma

A total of 93 paired-test results were compared. The mean HCV viral load measurement for PSC capillary was 6.127 log_10_ IU/mL, while the mean HCV viral load measurement for EDTA plasma was 6.126 log_10_ IU/mL.

**Fig. 1:**
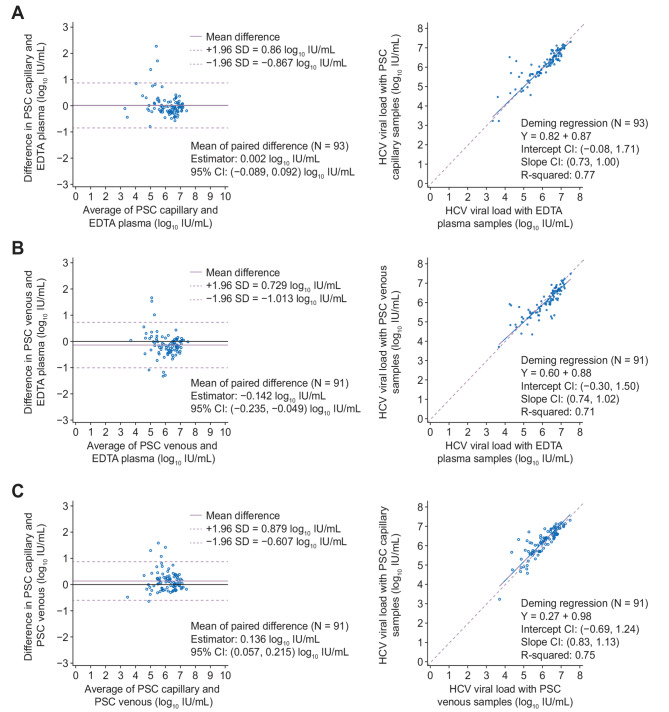
Bland–Altman analysis (left), and Deming regression (right) to assess comparability between (**A**) PSC capillary and EDTA plasma and (**B**) PSC venous and EDTA plasma samples, and (**C**) PSC capillary and PSC venous. PSC capillary represents Cobas PSC spotted with capillary blood (collected via finger prick); PSC venous represents Cobas PSC spotted with venous blood (collected in an EDTA tube). HCV, hepatitis C virus; PSC, plasma separation card.

Results of Bland–Altman analysis (Table 2a; Fig. 1a) showed similar HCV viral loads between PSC capillary and EDTA plasma, with a mean paired difference (95% CI) of 0.002 log_10_ IU/mL (− 0.089, 0.092). Approximately 95% of the paired samples had viral loads difference between − 0.857 log_10_ to 0.860 log_10_ IU/mL. Results of Deming regression analysis for the PSC venous and EDTA plasma samples showed no statistically significant constant difference in HCV viral load between the two sample matrices (intercept estimate of 0.82 [with 95% CI including 0]) (Fig. 1a). The slope estimate was 0.87 (with the 95% CI including 1), indicating no statistically significant proportional difference in HCV viral load between the two sample matrices (Table 2b; Fig. 1a).

**Table 2:** Comparability of HCV RNA results between different sample matrices by (a) Bland–Altman analysis, and (b) Deming regression.

(a) Bland-Altman: sample matrices compared	No. of paired samples	HCV RNA VL (log_10_ IU/mL)				
		Mean of PSC capillary	Mean of EDTA plasma	Mean (95% CI) of paired difference		
PSC capillary and EDTA plasma	93	6.127	6.126	0.002 (− 0.089, 0.092)		
PSC venous and EDTA plasma	91	6.042	6.184	− 0.142 (− 0.235, − 0.049)		
PSC capillary and PSC venous	91	6.178	6.042	0.136 (0.057, 0.215)		

(b) Deming: sample matrices compared	No. of paired samples	Parameter	Parameter estimate	Standard error	95% CI	R^2^
PSC capillary and EDTA plasma	93	Intercept	0.82	0.45	(− 0.08, 1.71)	0.77
		Slope	0.87	0.07	(0.73, 1.00)	
PSC venous and EDTA plasma	91	Intercept	0.60	0.45	(− 0.3, 1.50)	0.71
		Slope	0.88	0.07	(0.74, 1.02)	
PSC capillary and PSC venous	91	Intercept	0.27	0.49	(− 0.69, 1.24)	0.75
		Slope	0.98	0.08	(0.83, 1.13)	

The Deming regression analysis was based on paired Cobas HCV results falling within the overlapping linear range of EDTA plasma and PSC capillary samples (880 IU/mL to 1.00E + 08 IU/mL). Standard error and the 95% CIs for the Deming regression parameters were estimated using the jackknife method. PSC capillary represents Cobas PSC spotted with capillary blood (collected via finger prick); PSC venous represents Cobas PSC spotted with venous blood (collected in an EDTA tube). HCV, hepatitis C virus; PSC, plasma separation card; VL, viral load.

#### PSC venous and EDTA plasma

A total of 91 paired-test results were compared. The mean HCV viral load measurement for PSC venous was 6.042 log_10_ IU/mL, while the mean HCV viral load measurement for EDTA plasma was 6.184 log_10_ IU/mL.

 Bland–Altman analysis indicated that HCV RNA levels measured from PSC venous samples were slightly lower, on average, than those from EDTA plasma samples, with a mean paired difference (95% CI) of − 0.142 log_10_ IU/mL (− 0.235, − 0.049; Table 2a; Fig. 1b). For approximately 95% of the paired samples, the HCV viral load differences between the two matrices fell within the range of − 1.013 log_10_ IU/mL to 0.729 log_10_ IU/mL.

Results of Deming regression analysis for the PSC venous and EDTA plasma showed no statistically significant constant difference between these two sample matrices (intercept estimate of 0.60 [with the 95% CI including 0]), and no statistically significant proportional difference (slope estimate of 0.88 [with the 95% CI including 1]; Table 2b; Fig. 1b).

#### PSC capillary and PSC venous

A total of 91 paired-test results were compared. The mean HCV viral load for PSC capillary was 6.178 log_10_ IU/mL, while for PSC venous it was 6.042 log_10_ IU/mL.

Bland–Altman analysis of PSC capillary and PSC venous results showed a mean paired difference (95% CI) of 0.136 log_10_ IU/mL (0.057, 0.215; Table 2a; Fig. 1c), with HCV viral loads in PSC capillary being slightly higher, on average than PSC venous. For approximately 95% of the paired samples, the HCV viral load differences between the two matrices fell within the range of − 0.607 log_10_ to 0.879 log_10_ IU/mL.

Deming regression analysis for the comparison between PSC capillary and PSC venous showed no significant systematic difference between the two PSC sample types (intercept estimate of 0.27 [with the 95% CI including 0], and a slope estimate of 0.98 [with the 95% CI including 1; Table 2b; Fig. 1c]).

### Diagnostic accuracy

Clinical sensitivity and specificity at the HCV RNA threshold of 1000 IU/mL for Cobas HCV for PSC capillary compared with EDTA plasma were 100% (93/93; 95% CI 96.0, 100.0) and 100% (130/130; 95% CI 97.1, 100.0), respectively. PPV (95% CI) and NPV (95% CI) were 100.0% (93/93; 96.0, 100.0) and 100.0% (130/130; 97.1, 100.0; Table 3a).

**Table 3:** Clinical sensitivity and specificity at the HCV RNA threshold of 1000 IU/mL for (a) PSC capillary vs. EDTA plasma, and (b) PSC venous vs. EDTA plasma.

(a) Cobas HCV: PSC capillary result	Cobas HCV EDTA plasma result		Total
	≥ 1000 IU/mL	< 1000 IU/mL	
≥ 1000 IU/mL	93	0	93
< 1000 IU/mL	0	130	130
Total	93	130	223
Clinical sensitivity [n/N] (95% CI)	100.0% [93/93] (96.0, 100.0)		
Clinical specificity [n/N] (95% CI)	100.0% [130/130] (97.1, 100.0)		
Total percentage agreement [n/N] (95% CI)	100.0% [223/223] (98.3, 100.0)		
PPV [n/N] (95% CI)	100.0% [93/93] (96.0, 100.0)		
NPV [n/N] (95% CI)	100.0% [130/130] (97.1, 100.0)		

(b) Cobas HCV: PSC venous result	Cobas HCV EDTA plasma result		Total
	≥ 1000 IU/mL	< 1000 IU/mL	
≥ 1000 IU/mL	91	0	91
< 1000 IU/mL	2	131	133
Total	93	131	224
Clinical sensitivity [n/N] (95% CI)	97.8% [91/93] (92.5, 99.4)		
Clinical specificity [n/N] (95% CI)	100.0% [131/131] (97.2, 100.0)		
Total percentage agreement [n/N] (95% CI)	99.1% [222/224] (96.8, 99.8)		
PPV [n/N] (95% CI)	100.0% [91/91] (95.9, 100.0)		
NPV [n/N] (95% CI)	98.5% [131/133] (94.7%, 99.6%)		

PSC capillary represents Cobas PSC spotted with capillary blood (collected via finger prick); PSC venous represents Cobas PSC spotted with venous blood (collected in an EDTA tube). HCV, hepatitis C virus; NPV, negative predictive value; PPV, positive predictive value; PSC, plasma separation card.

For PSC venous compared with EDTA plasma in combination with Cobas HCV, clinical sensitivity and specificity at the HCV RNA threshold of 1000 IU/mL were 97.8% (91/93; 95% CI 92.5, 99.4) and 100% (131/131; 95% CI 97.2, 100.0), respectively. PPV (95% CI) and NPV (95% CI) were 100.0% (91/91; 95.9, 100.0) and 98.5% (131/133; 94.7, 99.6; Table 3b).

## Discussion

Detection and quantification of HCV RNA typically requires venous blood draw, and access to cold-chain sample storage and transportation for testing at centralized laboratory facilities. The results presented in this study demonstrated that when used in combination with Cobas HCV, Cobas PSC (both PSC capillary and PSC venous), compared with conventional EDTA plasma, had good diagnostic accuracy for HCV RNA detection at the HCV RNA threshold of 1000 IU/mL. The study findings also showed that Cobas PSC and plasma EDTA sample types yield comparable quantitative HCV RNA results.

Previous studies have described the value of DBS for specimen collection in the diagnosis of viral infections (e.g., HIV, hepatitis B virus and HCV)^18,30–33^. However, studies evaluating the performance of DBS have reported a higher LOD than that observed with plasma, and variable DBS performance depending on sample storage conditions^16,18,19,34–36^. The results of analytical performance evaluation studies that were conducted in parallel with the current clinical study determined an LOD for HCV RNA using Cobas PSC samples of 534.4 IU/mL and LLOQ of 880 IU/mL, indicating that HCV RNA can be reliably detected in specimens collected using Cobas PSC in individuals with HCV viral loads at a threshold of 1000 IU/mL. Results of the current analyses showed similar HCV viral load results for PSC capillary and EDTA plasma; however, HCV RNA levels measured from PSC venous samples were slightly lower on average than those from EDTA plasma samples. Yet, most of the difference fell within a narrow range, and overall, HCV RNA titers from PSC and EDTA plasma showed good comparability.

The overall comparability of HCV viral load measurements demonstrated in the current study between PSC and EDTA plasma sample types is in contrast to other capillary-based sample collection methods, such as DBS. Studies have shown HCV RNA titer differences from − 1.6 log_10_ IU/mL up to − 3.0 log_10_ IU/mL with DBS when compared with EDTA plasma or serum samples, depending on the HCV RNA assay used^16,18,37^. Previous studies comparing Cobas PSC specimens with EDTA plasma for HCV RNA quantification have reported HCV RNA titer differences of − 1.1 log_10_ IU/mL for PSC capillary^26^, and differences of − 1.64 log_10_ IU/mL and − 2.65 log_10_ IU/mL for PSC venous^14,22^. Of note, the results of all of the studies summarized above reported considerably lower HCV RNA titers for the DBS or PSC sample matrices compared with the serum or plasma reference samples. In the present study, comparable HCV viral load measurements were observed for different sample types (i.e., HCV viral load differences of up to − 0.14 log_10_ IU/mL [95% CI − 0.235, − 0.049] for PSC venous vs. EDTA plasma), even at higher HCV viral loads (e.g., 6 log_10_ IU/mL); this may be explained by the use of an assay-specific analysis package for HCV quantification on the PSC, which applies data analysis rules specific to this assay, and by high-quality PSC training of laboratory personnel that was included as part of a controlled clinical study environment. As part of the study initiation, the sponsor provided in-person training to all site members involved at each site for the sample collection as well as for the pre-analytical steps, ensuring that all critical aspects were addressed.

The comparability of quantitative HCV RNA results between PSC capillary and PSC venous indicates that Cobas PSC sample collection may be performed with either sample type by personnel with sufficient Cobas PSC training. Use of Cobas PSC may facilitate more convenient and flexible sample collection, depending on the local infrastructure and personnel (e.g., where there is no access to appropriate sample storage or phlebotomists), clinical situation (e.g., in individuals with limited venous access, such as PWID), or patient preference^13,22,36^. Additionally, the concurrently conducted analytical performance evaluation studies indicated that Cobas PSC provided a suitable matrix for storage and transportation of samples for HCV testing under extreme conditions (i.e., for up to 28 days at 18–45 °C and up to 85% relative humidity; Supplementary Table S2 online). This contrasts with studies on DBS that have shown degradation of HCV RNA during transportation or storage^35^.

WHO guidelines conditionally recommend the use of DBS specimens and refer to manufacturers’ protocols for DBS and PSC specimen collection in resource-poor settings and in persons with poor venous access, including drug treatment programs and prisons^10^. Based on the available evidence, Cobas PSC supports the WHO 2022 guideline recommendations on simplified HCV service delivery by facilitating decentralization of sample collection and offering the potential for integrated testing (i.e., cross-technology [serology and NAAT], multiple pathogens) as each PSC contains three distinct sample collection spots, each of which may be used for testing different parameters^9,10^.

The good diagnostic accuracy of Cobas PSC when used in combination with Cobas HCV observed in the present study demonstrates the clinical utility of Cobas PSC for correctly classifying HCV RNA test results below or above the threshold of 1000 IU/mL. There is general consensus that most untreated patients with HCV infection, as well as 95% of patients with detectable viremia 12 weeks after completing antiviral treatment (the recommended treatment endpoint), have HCV viral loads exceeding 1000 IU/mL^8,10^. In line with WHO and EASL guideline recommendations, methods such as Cobas HCV PSC that can detect viral HCV RNA levels using 1000 IU/mL thresholds are sufficient for clinical decision making in the vast majority of individuals^8,9^. Therefore, Cobas PSC may be suitable as an alternative method for HCV diagnosis, or as endpoint of therapy, where sensitive HCV RNA assays are not available and/or not affordable to detect viremic HCV infection or assess whether treatment has been successful^8,9,23^.

The study has several limitations. Subjects were enrolled from three sites in Spain and might not be representative of the broader population for whom HCV testing may be considered important (e.g., individuals from low-to-middle-income countries or specific settings [marginalized populations] in high-income countries). It is not possible to draw any conclusions regarding the performance of the Cobas PSC in detecting HCV viral loads of < 1000 IU/mL, as only a few samples with HCV viral loads between 15 IU/mL (LOD for plasma) and 1000 IU/mL, as well as between 1000 IU/mL and 10,000 IU/mL, were included in the study. The study’s findings further highlight the clinical relevance of the Cobas PSC for HCV viral load testing and suggest that Cobas PSC may provide a valuable tool for various clinical settings, potentially helping to broaden access to testing and to reduce the number of undiagnosed infected individuals in remote and under-resourced regions. However, studies with a larger sample size are required to confirm and allow generalization of these results, including a head-to-head comparison of the performance of DBS and PSC sample types for HCV RNA testing. In the absence of HCV-specific studies, a modeling study in HIV has reported that partial use of dried specimens (DBS and PSC) improves access to viral load testing for patients, at the lowest cost per correct result^38^. However, further exploration of the cost-effectiveness of Cobas PSC used for Cobas HCV testing is required.

## Conclusions

The findings of this study indicate that Cobas PSC provides a useful alternative to EDTA plasma for HCV RNA detection and quantification, with results demonstrating the comparability of quantitative HCV RNA results between these sample types. At the HCV RNA threshold of 1000 IU/mL, Cobas PSC in combination with Cobas HCV showed good diagnostic accuracy when compared with EDTA plasma. The use of Cobas PSC may help to extend access to central laboratory performance of HCV testing in remote and under-resourced regions where there is no access to venipuncture or sample refrigeration, as well as among individuals with limited venous access.

## Supplementary Information

Below is the link to the electronic supplementary material.

Supplementary Material 1

## Data Availability

Reasonable requests for datasets generated during and/or analyzed during the current study may be directed to [rotkreuz.datasharingrequests@roche.com](/cdn-cgi/l/email-protection).
