# Supplementary material for: HCV RNA quantification using the Cobas Plasma Separation Card as an alternative to EDTA plasma: a prospective multicenter study
Source: Sci Rep. 2026 Apr 20;16:18497. doi: 10.1038/s41598-026-45854-x (PMC13265737; doi:10.1038/s41598-026-45854-x)
Supplement: Supplementary file 1 — Supplementary Material 1 [file 41598_2026_45854_MOESM1_ESM.docx]

**Supplementary information**

HCV RNA quantification using the Cobas Plasma Separation Card as an alternative to EDTA plasma: A prospective multicenter study

Ana Fuentes López*, Ariadna Rando Segura*, Raquel Carracedo*, Aline Kessler, Adolfo de Salazar González, Sirak Zenebe Gebreab, Esther Molina, Noelia Parajó, Sara Pereira, Judit Romero Vico, Elena Ruiz Escolano, Marta Vila, Laura Viñuela González, Ana Carrasco Durán, Antonio Aguilera^†^, Federico García^†,ǂ^, Maria Buti^†,ǂ^

*Shared first authorship

^†^Shared last authorship

^ǂ^Corresponding authors

**Table of Contents**

| **Section** | **Page number** |
| --- | --- |
| **Methods** | 2 |
| *Analytical technical performance of the  Cobas PSC* | 2 |
| **Results** | 3 |
| **Supplementary Table 1.** LOD and LLOQ of   Cobas HCV when combined with Cobas  PSC | 3 |
| **Supplementary Table 2**. Clinical specimen   stability | 4 |
| **Supplementary Table 3:** Summary of study  population and analysis population  breakdown per site | 5 |
| **Supplementary Table 4**: Medical history  characteristics of study and analysis  population | 6 |
| **Supplementary Table 5.** Summary of HCV   viral load measurement groups based on  quantitative detection of HCV RNA in EDTA  plasma and HCV antibody test status | 8 |

**Supplementary methods**

*Analytical technical performance of the Cobas*® *PSC*

- The limit of detection (LOD) and lower limit of quantification (LLOQ) of Cobas PSC used with Cobas HCV were determined by Roche Diagnostics International Ltd as part of internal analytical performance evaluation studies conducted at the same time as this clinical study was conducted. Values from the analytical studies were applied to the analysis of the clinical study data.
- For determination of the LOD, an HCV-positive clinical specimen was used to prepare three independent dilution series that each included six concentration levels plus blank. The panels were spotted onto Cobas PSC from three different lots and tested with one Cobas HCV lot on Cobas 6800/8800 and 5800 Systems. The PROBIT value at 95% probability was determined for each PSC lot and all PSC lots combined.
- LLOQ and upper (U)LOQ values were calculated using a linearity panel with 11 concentration levels (range: 600 IU/mL to 1.0E+08 IU/mL). For each concentration, twelve replicates were spotted onto PSC from three different lots and tested on Cobas 6800 and 5800 Systems.
- The stability of dried plasma spots collected using the Cobas PSC was assessed by preparing five HCV-positive panels with a concentration of approximately five times the LLOQ (i.e., 4,400 IU/mL).
- For each different time point and condition being tested, three replicates of each panel were spotted onto three different Cobas PSC; the Cobas PSC were stored under various conditions (−10°C to 45°C, with/without separator, up to 85% humidity; **Supplementary Table S2**).
- The Cobas PSC were processed for testing with Cobas HCV on the Cobas 6800 system at the following five timepoints: immediate processing (t0), 15, 28/29, 58, and 85/86 days.

**Supplementary results**

**Supplementary Table S1.** LOD and LLOQ of Cobas HCV when combined with Cobas PSC

| **Parameter** | **Value (95% CI)** |
| --- | --- |
| LOD, IU/mL | 534.4 (460.0, 648.3) |
| LLOQ, IU/mL | 879 |
| Quantification linear range, IU/mL | 8.80E+02 to 1.0E+08 |

HCV, hepatitis C virus; LOD, limit of detection; LLOQ, lower limit of quantification; PSC, plasma separation card.

**Supplementary Table S2**. Clinical specimen stability

| **Storage time** | **Storage conditions** | **Layer** | **Stability demonstrated** |
| --- | --- | --- | --- |
| 16 days (T1) | +45°C, 85% rH (card transport) | With layer | 28 days transport stability at 18–45°C and up to 85% humidity |
| 29/30 days (T2) | +45°C, 85% rH (card transport) | With layer |  |
| 58/59 days (T3), 29 days after T2 | −15 to −20°C (card storage) | With layer | 56 days storage stability with and without layer separation at: ≤−10°C  2 to 8°C  18 to 30°C |
|  | −15 to −20°C (card storage) | No layer |  |
|  | 2 to 8°C (card storage) | With layer |  |
|  | 2 to 8°C (card storage) | No layer |  |
|  | 30°C (card storage) | With layer |  |
|  | 30°C (card storage) | No layer |  |
| 86/87 days (T4), 57 days after T2 | −15 to −20°C (card storage) | With layer |  |
|  | −15 to −20°C (card storage) | No layer |  |
|  | 2 to 8°C (card storage) | With layer |  |
|  | 2 to 8°C (card storage) | No layer |  |
|  | 30°C (card storage) | With layer |  |
|  | 30°C (card storage) | No layer |  |

PSC, plasma separation card; rH, relative humidity.

**Supplementary Table S3:** Summary of study population and analysis population breakdown per site

|  | **Barcelona** | **Granada** | **Santiago** | **Total** |
| --- | --- | --- | --- | --- |
| Enrolled subjects | 115 | 102 | 82 | 299 |
| HCV RNA detected | 54 | 29 | 25 | 108 |
| HCV RNA not detected | 56 | 67 | 56 | 179 |
| Other HCV RNA (<titer minimum) | 5 | 6 | 1 | 12 |
| Analysis population | 71 | 89 | 64 | 224 |
| HCV RNA detected | 54 | 28 | 23 | 105 |
| HCV RNA not detected | 17 | 61 | 41 | 119 |
| Subjects excluded from the analysis population | 44 | 13 | 18 | 75 |
| Not meeting inclusion criteria |  |  |  |  |
| Anti-HCV positive and HCV RNA TND | 35 | 1 | 8 | 44 |
| No capillary blood was collected | - | 3 | 1 | 4 |
| No EDTA blood was collected | - | 3 | - | 3 |
| ICF was not signed during enrolment | - | - | 5* | 0 |
| Plasma <15 IU/mL | 5 | - | 1 | 6 |
| Familiarization phase | 4 | 5 | 3 | 12 |
| Patient refused to participate | - | 1 | - | 1 |

HCV, hepatitis C virus; ICF, informed consent form; TND, target not detected.

**Supplementary Table S4**: Medical history characteristics of study and analysis population

| **Characteristics** | **Enrolled population** | **Analysis population^a^** |
| --- | --- | --- |
| **Total, N** | 299 | 224 |
| Previous HCV infection status, n (%) |  |  |
| Negative | 125 (41.8) | 85 (37.9) |
| Positive | 134 (44.8) | 103 (46.0) |
| Suspicion positive | 4 (1.3) | 3 (1.3) |
| Not reported | 35 (11.7) | 33 (14.7) |
| Anti-HCV therapy received, n (%) |  |  |
| No | 197 (65.9) | 163 (72.8) |
| Yes | 50 (16.7) | 13 (5.8) |
| Not reported | 51 (17.0) | 48 (21.4) |
| Anti-HCV treatment regimen used, n (%)^b^ |  |  |
| Sofosbuvir/velpatasvir | 40 (13.4) | 12 (5.4) |
| Sofosbuvir/velpatasvir/voxilaprevir | 0 (0.0) | 0 (0.0) |
| Grazoprevir/elbasvir | 1 (0.3) | 0 (0.0) |
| Glecaprevir/pibrentasvir | 6 (2.0) | 1 (0.4) |
| Other | 2 (0.7) | 0 (0.0) |
| Unknown | 1 (0.3) | 0 (0.0) |
| Patient received anti-HCV treatment in past 2 weeks, n (%)^b^ |  |  |
| Yes | 15 (5.0) | 11 (4.9) |
| No | 35 (11.7) | 2 (0.9) |
| HCV antibody test status |  |  |
| Negative | 134 (44.8) | 119 (53.1) |
| Positive | 152 (50.8) | 103 (46.0) |
| Other^c^ | 2 (0.7) | 2 (0.9) |
| Not applicable | 1 (0.3) | 0 (0.0) |

^a^Analysis population includes samples that contributed for at least one of the study objectives; ^b^Numbers are based on subjects who received anti-HCV therapy; ^c^Anti-HCV antibody test was not performed due to insufficient sample volume; however, the subject's previous HCV infection status was confirmed to be positive.
HCV, hepatitis C virus.

**Supplementary Table S5.** Summary of HCV viral load measurement groups based on quantitative detection of HCV RNA in EDTA plasma and HCV antibody test status

| **Viral load group** | **HCV viral load results in EDTA plasma** | **Anti-HCV status** | **Planned samples** | | **Actual samples** |
| --- | --- | --- | --- | --- | --- |
|  |  |  | **Samples across groups (n)** | **Samples per group (n)** | **Total samples (n)** |
| 1 | Target not detected | Negative | 100 | 100 | 119 |
| 2 | ≥titer min (LLOQ VL EDTA plasma) to <LLOQ VL PSC | Positive | 25 | 10 | 12 |
| 3 | ≥LLOQ VL PSC <1,000 IU/mL | Positive |  | 5 | 0 |
| 4 | ≥1,000 IU/mL to <10,000 IU/mL | Positive | 120 | 15 | 3 |
| 5 | ≥≤1E+04 IU/mL to 1E+06 IU/mL | Positive |  | 15 | 31 |
| 6 | ≥1E+06 IU/mL to ≥1E+08 IU/mL | Positive |  | 15 | 59 |

Note: Groups 3–6 were eligible for matrix equivalency analysis along the overlapping linear range of EDTA plasma and PSC sample types. Group 1–3 were eligible for clinical specificity and groups 4–6 were eligible for clinical sensitivity analysis.
Anti-HCV, antibodies to HCV; HCV, hepatitis C virus; LLOQ, lower limit of quantification; N, number of samples; PSC, plasma separation card; ULOQ, upper limit of quantification; VL, viral load.
